# Methicillin-Resistant *Staphylococcus aureus* breast abscesses: Risk factors and outcomes from a tertiary care center of a lower-middle-income country

**DOI:** 10.12669/pjms.42.(11AASC).15658

**Published:** 2026-04

**Authors:** Narmeen Asif, Sana Zeeshan, Iffat Khanum, Imran Ahmed

**Affiliations:** 1 Dr. Narmeen Asif, FCPS, Department of Surgery, Section of General Surgery, Aga Khan University Hospital, Karachi, Pakistan; 2 Dr. Sana Zeeshan, FCPS, FACS, MRBS, Department of Surgery, Aga Khan University Hospital, Karachi, Pakistan; 3 Dr. Iffat Khanum, FCPS, Department of Medicine, Section of Infectious Diseases, Aga Khan University Hospital, Karachi, Pakistan; 4 Dr. Imran Ahmed, FCPS, Section of Microbiology, Department of Pathology and Laboratory Medicine, Aga Khan University Hospital, Karachi, Pakistan

**Keywords:** Antibiotic resistance, Breast abscess, Lactational mastitis, MRSA, Methicillin-resistant Staphylococcus aureus, Ultrasound-guided aspiration

## Abstract

**Background and Objectives:**

Breast abscesses are a common complication of mastitis, traditionally caused by methicillin-sensitive *Staphylococcus aureus* (MSSA). Recent global reports, however, indicate a rising prevalence of methicillin-resistant *Staphylococcus aureus* (MRSA), with limited data from South Asia. The objective of this study was to determine the prevalence, risk factors, antibiotic susceptibility patterns, management strategies, and clinical outcomes of MRSA breast abscesses at a tertiary-care center in Pakistan.

**Methodology:**

A retrospective cross-sectional study was conducted at Aga Khan University Hospital, Karachi, from January 2010 to December 2024. All culture-positive breast abscesses with *S. aureus* growth were included. Patient demographics, clinical features, microbiological data, management, and outcomes were analyzed using descriptive statistics.

**Results:**

Of 366 culture-positive breast abscesses, 326 (89.0%) yielded *S. aureus*, of which 274 (74.8% out of total) were MRSA, representing 74.5% of all isolates. A marked temporal shift was observed: MSSA remained relatively common from 2010–2019, but only 14 MSSA cases occurred from 2020–2024, with MRSA becoming the dominant pathogen. The mean age of MRSA patients was 29.4 ± 7.1 years; 88.7% were lactational. Key risk factors included prior antibiotic exposure (63.0%), recent hospitalization (55.9%), diabetes (18.6%), and history of previous abscess (14.4%). All MRSA isolates were vancomycin-susceptible; resistance rates were low for clindamycin (11.7%) and trimethoprim–sulfamethoxazole (9.8%). Initial ultrasound-guided aspiration was performed in 60.2%, with 20.6% requiring subsequent incision and drainage. Empirical clindamycin was used in 53.6%. Complete resolution was achieved in all patients with documented follow-up, with a recurrence rate of 3.3%. Among lactational cases, 91.4% successfully resumed breastfeeding.

**Conclusion:**

This study reports one of the highest documented MRSA prevalence rates (74.5%) in breast abscesses worldwide, with a clear post-2020 dominance. Despite high MRSA burden, excellent outcomes were achieved with ultrasound-guided drainage and susceptibility-guided antibiotics. In high-prevalence settings, empirical therapy must cover MRSA, and routine culture with sensitivity testing is strongly recommended. Ultrasound-guided aspiration should remain first-line management, and continued breastfeeding is safe and encouraged after treatment.

## INTRODUCTION

Mastitis, an inflammation of breast tissue, has been commonly seen in post-partum lactating females, with an incidence of as high as 20% reported in the literature.^1^,^2^ However, it can also occur in non-lactating females and at any age.^3^ If it remains untreated, this often leads to the most common complication of breast abscess, which is a collection of pus in breast tissue^2^. Breast abscesses can also develop directly without preceding mastitis.^2^ *Staphylococcus aureus (S. aureus)* has been reported as the most common organism causing mastitis and even breast abscess in both lactating and non-lactating females, with incidence rising from 40-50% in puerperal mastitis to 67-84% in breast abscess.^1^,^3^-^6^ However, few studies have recently reported a rise in cases of breast abscess harbouring methicillin-resistant *Staphylococcus aureus* (MRSA) with incidence as high as 50% in lactating mothers.^3^ Other organisms besides *S. aureus* that have been implicated in breast abscesses are *Klebsiella pneumonia, Bacteroides, Pseudomonas aeruginosa, Streptococcus species, Fusobacterium species* and *Mycobacterium tuberculosis* with mixed and anaerobic organisms more commonly found in non-lactational breast abscess.^3^,^7^,^8^

The main risk factors identified for breast abscess in lactating females are nipple cracks, milk stasis, primiparity and breast engorgement, while smoking, diabetes, obesity, HIV, poor socioeconomic status and females of black ethnicity have been associated as risk factors for development of non-lactational breast abscess.^3^,^6^,^9^,^10^ MRSA is more commonly hospital acquired, but community-acquired MRSA accounts for nearly 19% of all breast abscess cases as per recent studies.^11^ MRSA signifies resistance to all β-lactam antibiotics such as the penicillins (methicillin, dicloxacillin, nafcillin, oxacillin, etc.) and the cephalosporins, making its treatment difficult with standard antibiotics.^12^ In addition, community acquired MRSA (CA-MRSA) carries a gene encoding a cytotoxin known as Panton-Valentine Leukocidin (PVL) that causes cell membrane lyses of human neutrophils leading to severe infections and prolonged hospital stay.^1^,^12^,^13^ The treatment of breast abscesses involves incision and drainage or image-guided aspiration, along with culture-directed antibiotics. However, because MRSA is resistant to many antibiotics, treatment options for MRSA-associated breast abscesses are limited to a few agents, such as trimethoprim–sulfamethoxazole, clindamycin, tetracycline, vancomycin, and linezolid.^11^,^14^ Recent studies have shown increasing resistance of MRSA to clindamycin and tetracycline resulting in treatment failures^2^. The rising prevalence of multidrug-resistant organisms in breast abscesses further highlights the need for ongoing surveillance and tailored antimicrobial strategies.^15^,^16^

Despite the global rise in MRSA breast abscesses, there is a paucity of data from South Asia, particularly Pakistan. Regional differences in bacterial epidemiology, antibiotic resistance, and healthcare practices necessitate local studies to inform effective management protocols. Reporting data from Pakistan is crucial to understanding the local burden, guiding empirical therapy, and contributing to the global effort to control MRSA-related morbidity in breastfeeding women.^15^,^16^

### Rationale:

With the rising incidence of MRSA breast abscesses worldwide and in Pakistan, this study focuses on evaluating cases of MRSA breast abscesses at our institution to identify potential risk factors, antibiotic susceptibility patterns, management strategies, and clinical outcomes. Detailed analysis of MRSA breast infections will help develop evidence-based institutional protocols for the effective management of MRSA breast abscesses in our population.

Our objective was to identify frequency, risk factors and antibiotic sensitivity patterns of MRSA in breast abscesses and to evaluate management in terms of intervention and choice of antibiotics along with its outcomes in MRSA breast abscess.

## METHODOLOGY

This was a cross-sectional study conducted at the Section of Breast Surgery, Department of Surgery, Aga Khan University Hospital, Karachi. Clinical data was retrospectively collected for all patients diagnosed with breast abscess caused by Methicillin Resistant or Methicillin Sensitive *S. aureus* between January 2010 and December 2024. Non-probability purposive sampling was employed to include eligible cases.

### Inclusion and Exclusion criteria:

All female patients who presented to the hospital through the emergency department, outpatient clinics, or inpatient services, and had a clinically and/or radiologically confirmed breast abscess with available culture and sensitivity reports were included. Patients with non-bacterial causes of breast abscess such as granulomatous mastitis, or carcinoma, those with incomplete medical records, or cases with mixed bacterial growth other than MRSA and MSSA were excluded.

### Data Collection:

Data was collected retrospectively from electronic medical records and physical files, radiology, and microbiology departments. Investigators used a predesigned proforma to record patient demographics, comorbidities, parity, lactational status, causative organisms and antibiotic sensitivities, empirical and culture-directed antibiotic therapy and duration, type of intervention, hospitalization requirement, time to abscess resolution, resumption of breastfeeding, and any treatment-related complications.

### Ethical considerations:

As this study involved retrospective review of existing medical records with no direct patient contact, exemption from the Ethical Review Committee of Aga Khan University Hospital was sought (ERC number 2025-5100-38270; dated October 20, 2025).

### Statistical analysis:

Statistical analysis was performed using SPSS version 24. Descriptive statistics were used to summarize the study population. Categorical variables, such as type of organism, clinical features, risk factors, interventions, antibiotic use, and outcomes, were expressed as frequencies and percentages. Continuous variables, such as age and duration of antibiotic therapy, were summarized using mean and standard deviation.

## RESULTS

A total of 366 female patients with culture-positive breast abscesses were included between January 2010 and December 2024. *S. aureus* was the predominant pathogen, isolated in 326 patients (89.0%). Of these *S. aureus* isolates, 274 (74.8 %) were MRSA and 52 (14.2 %) were MSSA. The remaining 40 patients (10.9%) grew other organisms, including Corynebacterium species, Mycobacterium tuberculosis, and mixed anaerobes.

A clear temporal shift toward MRSA predominance was observed. During the first decade (Jan 2010 to Dec 2019), MSSA remained relatively common, whereas in the subsequent five years period (Jan 2020 to Dec 2024) only 14 new MSSA cases were recorded compared with a sustained increase in MRSA isolates, resulting in MRSA accounting for most *S. aureus* breast abscesses in recent years.

Among the 274 patients with MRSA breast abscesses, the mean age at presentation was 29.4 ± 7.1 years. Most cases were lactational abscesses (88.7%, n = 243), while 31 patients (11.3%, n = 31) had non-lactational infections. The most common presenting symptoms were breast pain (96%, n = 263), erythema (89%, n = 244), nipple cracks/fissures (61%, n = 167), and fever (42%, n = 115). Abscesses were most frequently located in the upper outer quadrant (48%, n = 132), followed by the upper inner quadrant (22%, n = 60), lower outer quadrant (19%, n = 52), and lower inner quadrant (11%, n = 30).

Comorbidities and risk factors in the MRSA cohort included diabetes mellitus (18.6%, n = 51), prior antibiotic exposure within the preceding 3 months (63.0%, n = 173), and history of previous breast abscess (14.4%, n = 40). Recent hospitalization (within the preceding six months), mostly for childbirth, was documented in 55.9% (n = 153).

All MRSA isolates (100%) remained sensitive to vancomycin. Resistance rates to other agents were clindamycin 11.7% (n = 32), erythromycin 19.7% (n = 54), ciprofloxacin 13.9% (n = 38), and trimethoprim–sulfamethoxazole 9.8% (n = 27). Sensitivities for linezolid are not routinely reported at our institution.

**Table-I:** Microbiological profile and patient characteristics of all culture positive breast abscess patients.

Variable	Total Patients (n=366)
***Sex distribution***	
– Female	366 (100%)
***Organisms isolated***	
– MRSA	274 (74.8%)
– MSSA	52 (14.2%)
– Other organisms (e.g., Corynebacterium, TB, mixed anaerobes)	40 (10.9%)

**Table-II:** MRSA Breast Abscess: Clinical Characteristics, Management and Outcomes (n = 274).

Parameter	Value
Mean age (years)	29.4 ± 7.1 (Range 20- 68 years)
Type of abscess	
– Lactational	243 (88.7%)
– Non-lactational	31 (11.3%)
Common presenting features	
– Breast pain	263 (96%)
– Erythema	244 (89%)
– Nipple cracks	167 (61%)
– Fever	116 (42%)
Risk factors	
– Diabetes mellitus	51 (18.6%)
– Hospitalization within previous 6 months	153 (55.9%)
– Prior antibiotic use	173 (63%)
– Previous breast abscess	39 (14.4%)
Initial management	
– Ultrasound-guided aspiration	165 (60.2%)
– Primary incision & drainage (I&D)	109 (39.8%)
Need for escalation of treatment	
– Conversion from aspiration to I&D	24/165 (20.2%)
Antibiotics (empirical pre-culture)	
– Clindamycin	147 (53.6%)
– Amoxicillin-clavulanate	85 (31.0%)
– Linezolid	42 (15.3%)
Antibiotic resistance pattern	
– Vancomycin resistance	0 (0%)
– Clindamycin resistance	32 (11.7%)
– Erythromycin resistance	54 (19.7%)
– Ciprofloxacin resistance	38 (13.9%)
– TMP-SMX resistance	27 (9.8%)
Outcomes	
– Lost to followup	43/165 (26.0%)
– Complete clinical resolution	234 (100%)
– Mean time to improvement (days)	7.8 ± 2.6
– Multiple aspiration sessions required	72 (43.6%)
– Recurrence	9 (3.3%)
– Returned to breastfeeding (among lactational patients)	222 (91.4%)

Of the 274 MRSA patients, 165 (60.2%) were initially managed with ultrasound-guided aspiration and 109 (39.8%) underwent primary incision and drainage (I&D). Among those starting with aspiration, 34 patients (20.6% of aspiration group) required subsequent conversion to I&D, most commonly due to failed repeated aspirations, overlying skin necrosis, or imminent spontaneous rupture.

Empirical antibiotics prescribed before culture results were available were clindamycin (53.6%, n = 147), amoxicillin–clavulanate (31.0%, n = 85), and linezolid (15.3%, n = 42). After culture confirmation, antibiotic regimens were modified in 18 patients (6.6%) to align with susceptibility profiles. The mean duration of antibiotic therapy was 12 ± 4 days. Hospital admission was generally reserved for patients undergoing I&D; 54% of I&D patients (n = 59/109) were managed as same-day care without overnight stay.

Of the 165 patients initially treated with ultrasound-guided aspiration, 43 (26.0%) were lost to follow-up after the initial procedure and have no further outcome data. Complete clinical resolution was achieved in all patients with documented follow-up, with a mean time to clinical improvement of 7.8 ± 2.6 days. Multiple aspiration sessions were required in 72 patients (43.6% of the aspiration group). Recurrence (new abscess at the same site after initial resolution) occurred in 9 patients overall (3.3%, n = 9/274). Among lactational patients with MRSA (n = 243), 91.4% (n = 222) resumed breastfeeding after recovery. No significant medication-related adverse effects were observed in breastfed infants, apart from mild diarrhoea in 3 neonates (1.2% of lactational cases) whose mothers received clindamycin.

## DISCUSSION

This study documents an exceptionally high prevalence of MRSA in culture-proven breast abscesses at a single tertiary-care center in Pakistan, accounting for 74.5% of all isolates and 84.0% of *S. aureus* cases. A distinct temporal trend was observed: during the first decade (2010–2019), MSSA remained relatively common, whereas in next five years i.e. from 2020 to 2024, MRSA became the overwhelmingly dominant pathogen, with only 14 new MSSA cases identified in the most recent four-year period. This represents one of the highest reported MRSA rates in breast abscesses worldwide and underscores the rapidly evolving microbiology of this condition in our region.

Earlier studies from South Asia and the Middle East reported MRSA in 20–50% of breast abscesses^3^,^6^,^13^,^15^,^16^ while mid-2000s outbreaks in the United States reached 60–70%.^11^ More recent series from Malaysia and India have described rates approaching or exceeding 70%,^16^ bringing them closer to our findings. The sharp acceleration after 2019–2020 coincides with global reports of increased circulation of certain community-onset MRSA clones during and after the COVID-19 pandemic, possibly related to changes in healthcare-seeking behavior, antibiotic prescribing patterns, or infection-control practices.^17^,^18^ Although 55.9% of our patients had been hospitalized within the preceding six months (mostly for childbirth), the combination of young age, lactational status, and community-onset presentation in the majority supports a predominantly healthcare-associated/community-onset rather than purely nosocomial epidemiology.

The clinical profile of predominantly young lactating women with nipple fissures, recent delivery, prior antibiotic exposure (63%), and frequent postpartum hospitalization, aligns with well-established risk factors for puerperal mastitis and abscess.^2^,^5^ However, the near-complete replacement of MSSA by MRSA has rendered traditional beta-lactam-based empirical regimens ineffective in our setting. Clindamycin was the most frequently used empirical agent (53.6%), yet 11.7% of isolates were resistant, consistent with emerging clindamycin resistance reported from Pakistan and neighboring countries.^3^,^16^ All isolates remained susceptible to vancomycin, and resistance to trimethoprim–sulfamethoxazole was low (9.8%). Institutional data also demonstrate near-100% linezolid susceptibility among *S. aureus* isolates (including MRSA), making oral linezolid a reliable step-down or outpatient option where cost and availability permit.^19^

Ultrasound-guided aspiration as first-line therapy was successful in approximately 80% of patients who completed follow-up, with a low recurrence rate (3.3%). These results reinforce international evidence that minimally invasive drainage combined with effective antibiotics can replace Incision and drainage in most cases, even when MRSA is the causative pathogen.^7^,^10^,^14^ The 26% loss-to-follow-up after initial aspiration reflects a known challenge of outpatient-based management in private-sector low- and middle-income country (LMIC) settings and highlights the need for structured follow-up pathways.

Successful resumption of breastfeeding in 91.4% of lactational cases, with only minor infant side-effects, reaffirms that appropriately treated MRSA breast abscess is not a contraindication to continued lactation which is an important counselling point for both clinicians and mothers.^1^,^4^

### Limitations:

It was a single center study and since the institution does not have a dedicated database for breast abscess, data was collected from multiple sources (breast clinics, radiology, microbiology, HIMS) which may result in missing of approximately 10% of actual data.

## CONCLUSION

In settings with high MRSA prevalence such as ours, empirical therapy for breast abscess should include an MRSA-active agent (vancomycin, linezolid, or clindamycin with knowledge of local resistance patterns) until culture results are available. Routine culture and sensitivity testing of all drained breast abscesses is strongly recommended. First-line ultrasound-guided aspiration combined with targeted antibiotics remains highly effective and should be the standard of care. These findings challenge older guidelines that continue to recommend beta-lactam antibiotics as empirical therapy for lactational mastitis/abscess and support urgent revision of institutional and regional treatment protocols.

### Author’s Contribution:

**NA:** Conception and design of the study, data acquisition, interpretation, analysis and manuscript writing

**IK IA:** Data acquisition, interpretation, and manuscript writing

**SZ:** Conception and design of the study, overall supervision, and final responsibility for the integrity and accuracy of work.

All authors have approved the final version of the manuscript and agree to be accountable for all aspects of the work.
